# Neural networks with personalized training for improved MOLLI T_1_ mapping

**DOI:** 10.1186/s12880-025-01769-z

**Published:** 2025-07-01

**Authors:** Olympia Gkatsoni, Christos G. Xanthis, Sebastian Johansson, Einar Heiberg, Håkan Arheden, Anthony H. Aletras

**Affiliations:** 1 Laboratory of Computing, Medical Informatics and Biomedical – Imaging Technologies, School of Medicine, Aristotle University of Thessaloniki, Thessaloniki, Greece; 2 Clinical Physiology, Department of Clinical Sciences Lund, Lund University, Skåne University Hospital, Lund, Sweden; 3 Wallenberg Centre for Molecular Medicine, Lund University, Lund, Sweden

**Keywords:** Cardiac MRI, Deep learning, T_1_ mapping, MRI simulator

## Abstract

**Background:**

The aim of this study was to develop a method for personalized training of Deep Neural Networks by means of an MRI simulator to improve MOLLI native T_1_ estimates relative to conventional fitting methods.

**Methods:**

The proposed Personalized Training Neural Network (PTNN) for T_1_ mapping was based on a neural network which was trained with simulated MOLLI signals generated for each individual scan, taking into account both the pulse sequence parameters and the heart rate triggers of the specific healthy volunteer. Experimental data from eleven phantoms and ten healthy volunteers were included in the study.

**Results:**

In phantom studies, agreement between T_1_ reference values and those obtained with the PTNN yielded a statistically significant smaller bias than conventional fitting estimates (-26.69 ± 29.5ms vs. -65.0 ± 33.25ms, *p* < 0.001). For in vivo studies, T_1_ estimates derived from the PTNN yielded higher T_1_ values (1152.4 ± 25.8ms myocardium, 1640.7 ± 30.6ms blood) than conventional fitting (1050.8 ± 24.7ms myocardium, 1597.2 ± 39.9ms blood). For PTNN, shortening the acquisition time by eliminating the pause between inversion pulses yielded higher myocardial T_1_ values (1162.2 ± 19.7ms with pause vs. 1127.1 ± 19.7ms, *p* = 0.01 myocardium), (1624.7 ± 33.9ms with pause vs. 1645.4 ± 18.7ms, *p* = 0.16 blood). For conventional fitting statistically significant differences were found.

**Conclusions:**

Compared to T_1_ maps derived by conventional fitting, PTNN is a post-processing method that yielded T_1_ maps with higher values and better accuracy in phantoms for a physiological range of T_1_ and T_2_ values. In normal volunteers PTNN yielded higher T_1_ values even with a shorter acquisition scheme of eight heartbeats scan time, without deploying new pulse sequences.

## Background

Quantification of native myocardial T_1_ relaxation time has the potential to detect cardiomyopathies and has been suggested as a non-invasive tissue characterization method. Changes in native T_1_ values could potentially be used as a biomarker with potential prognostic, diagnostic and therapeutic significance [1–3]. Native T_1_ maps have been shown to detect both ischemic [3–6] and non-ischemic [2, 7–13] cardiomyopathies as well as characterize iron overload [14]. Also, changes in the myocardium over time may be assessed with T1 mapping [4].

Myocardial T_1_-mapping remains a challenging task due to the limitations imposed by the cardiac and respiratory motion [15]. Prior to clinical use, T_1_ quantitative methods should be assessed and compared in terms of their accuracy and precision [16]. The precision of T_1_-mapping affects the detection of abnormal myocardial tissue [6] while the accuracy of these measurements affects the ability to compare with other sites as well as with the true underlying values [16].

MRI simulators have been used to address the measurement problems with T_1_ mapping techniques [17]. However, they have been utilized within a limited scope in cardiovascular clinical applications. Parallel Simulations for Quantifying Relaxation Magnetic Resonance constants (SQUAREMR) [18, 19] has been introduced for obtaining quantitative CMR tissue information from clinical mapping pulse sequences. This method utilizes a database of realistic simulated Modified Look-Locker Inversion recovery (MOLLI) signals that integrate the individual ECG triggers for each study and aims to find the database entry closest to the acquired MRI signal. Cardiac Magnetic Resonance Fingerprinting (cMRF) [20–23] takes a similar approach, generating a new dictionary which incorporates each specific subject’ s heart rate, by means of a research custom designed pulse sequence. cMRF is a rapidly developing technique [24] that enables a single protocol for quantifying additional tissue properties by reducing the number of breath-held scans [25]. BlessPC [26] has also been presented as a method for more accurately quantifying myocardial T_1_. It uses a FLASH-based MOLLI at 3.0 Tesla and is based on Bloch equation simulations with slice profile correction. BlessPC simulations consider a range of heart rates with increments and the results show reduced sensitivity to heart rate variations.

Machine learning has been proposed as an alternative to standard curve-fitting techniques for quantitative CMR with MOLLI. MyoMapNet [27] utilized deep learning neural networks for T_1_ mapping from only five or four MOLLI images, while preserving the accuracy of the original MOLLI acquisitions for 5(3)3 and 4(1)3(1)2, for native and post-contrast respectively (where numbers outside of the parentheses represent number of heartbeats following an inversion pulse where data are acquired and numbers inside the parentheses represent recovery pause intervals in terms of number of heartbeats). Simulations are generated for a fixed heart rate for MyoMapNet. DeepBless [28] was also introduced with deep learning Bloch equation simulations that consider a randomly sampled perspective of heart rates within a predefined range, yielding rapid calculation of myocardial T_1_ values similar to that of BlessPC.

More recently, improvements in myocardial T_1_ measurement accuracy with MyoMapNet [29] were made possible by training the neural network both with numerically simulated data as well as with phantom data and integration of a range of simulated heart rates. In addition, acceleration in MyoMapNet [30] was shown by training the neural network with a combination of native and post-contrast T_1_-weighted ECG-triggered images collected with a single inversion pulse in four heartbeats. The inclusion of individual ECGs in training data in that study resulted in slightly higher native T_1_ values compared to MOLLI 5(3)3 for both myocardium and blood. Heart rate changes or shorter T_1_ values can influence the results. Scanner vendor and field strength information [31] was also included to MyoMapNet for extending the applicability of the method. Last, other approaches, not based on MOLLI, have been introduced for accurately estimating cardiac T_1_ and T_2_ [32].

Neural networks in cMRF have been applied to speed up the dictionary generation [33] without significantly changing the reconstruction. A self-supervised deep learning reconstruction that employs deep image priors, DIP-MRF [34] was developed for denoising and mitigating aliasing artifacts in 5 heartbeat breath-held time scans. cMRF has developed as a promising research approach for simultaneous T_1_ and T_2_ mapping that has limitations with respect to computational complexity and time required. Also, more standardization and validation is needed before it is applied in the clinics.

There is still room in terms of improving MOLLI T_1_ mapping accuracy, without sacrificing precision. In the clinics, the MOLLI pulse sequence is uniquely applied to each patient by triggering the inversion pulses according to his/her unique ECG at the time of data acquisition. In this study, we hypothesized that utilizing a neural network with personalized training with the volunteer’s unique ECG trigger data could help towards better MOLLI accuracy without sacrificing precision. Incorporating the volunteer’s specific unique ECG data into the network’s training removes the ECG from being a variable that the neural network has to compensate for. MR data from Bloch simulations run with the volunteer’s unique ECG could potentially be used to train a neural network that can be used to estimate T_1_ only for this specific volunteer. Training a neural network on a patient-by-patient basis is a paradigm shift since in this case the network is trained to yield an output based on data acquired from only one specific patient rather than the usual approach where a network is trained to yield an output based on data acquired from many patients.

Therefore, the aim of this study was to improve accuracy without sacrificing precision with MOLLI 5(3)3 for both native myocardial and blood T_1_ estimates by developing a machine learning approach, as an alternative to the standard curve-fitting used for T_1_ map generation, which would be based on Deep Neural Networks trained for each specific volunteer with simulated MR data that take into account his/her ECG at the time of the MOLLI acquisition. If successful, this would also potentially allow a faster acquisition MOLLI scheme, such as the 5(0)3, to be applied.

We hypothesized that T_1_ estimation with this personalized training deep learning network approach would be more accurate than conventional fitting for (1) a physiological range of T_1_ and T_2_ values, (2) variable heart rates during the scan, (3) a shorter acquisition MOLLI scheme with just eight heartbeats scan time i.e., 5(0)3.

## Methods

### Personalized training neural network (PTNN) overview

The proposed method for T_1_ mapping was based on a deep learning neural network which was trained with simulated MOLLI signals. The simulated signals were generated for each research volunteer based on the MOLLI pulse sequence with the exact inversion times (TIs) used in the MRI scanner, which depended on the ECG of the volunteer. The simulated MOLLI signals were generated with a parallel Bloch equation simulation platform (CoreMRI) ([35–37] for a physiological range [38, 39] of T_1_ values and T_2_ values. This personalized training neural network was then used to estimate native T_1_ values from MOLLI images acquired from each healthy volunteer. Since the network was individually trained for each volunteer and his/her ECG trigger times, training times had to be relatively short.

**Fig. 1:**
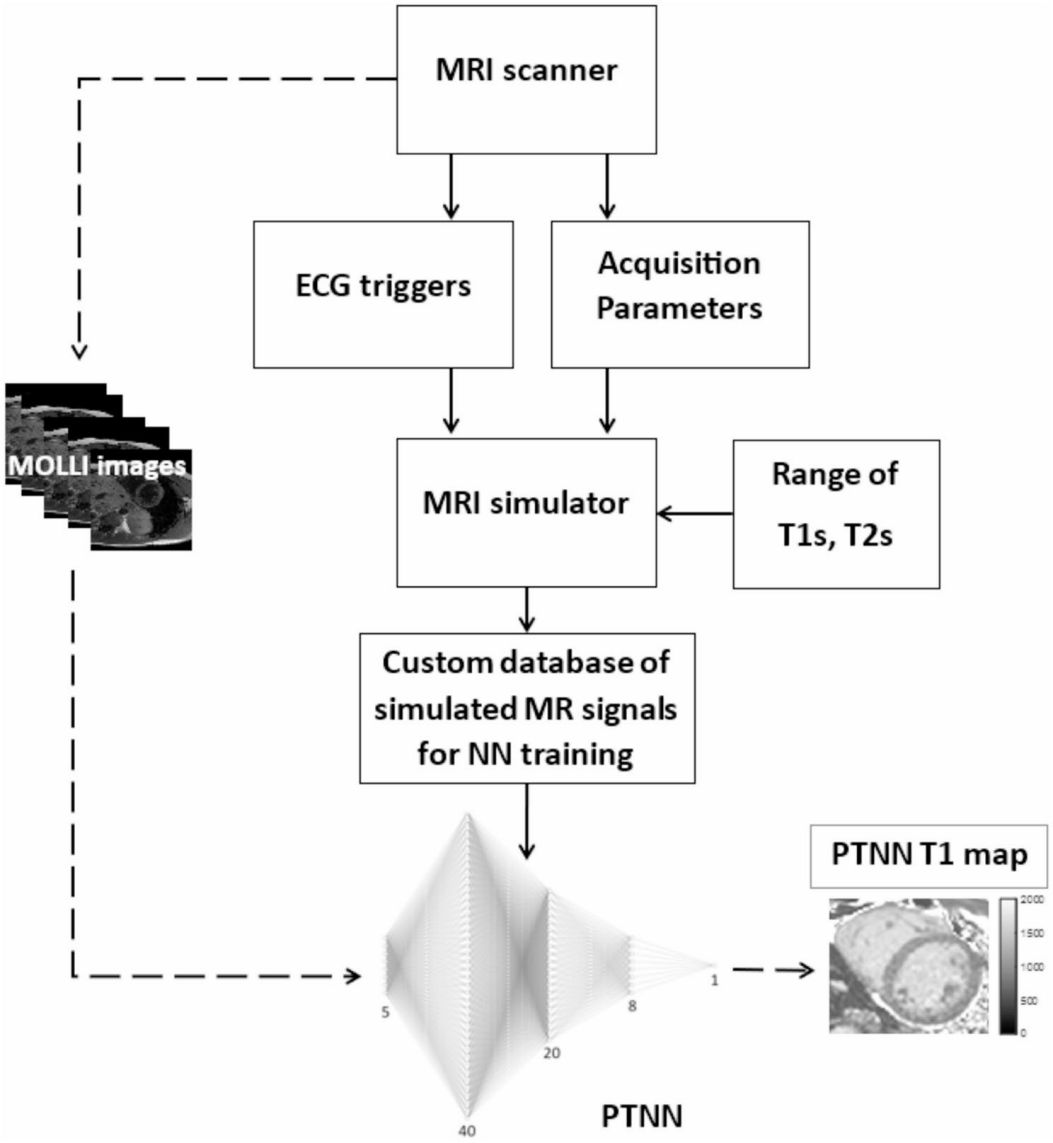
Block diagram for personalized-training and using the neural network for estimating T_1_ values with MOLLI. Personalized-training (solid lines) was performed by simulating the MOLLI pulse sequence with its parameters and the physiological ECG triggers so as to generate a customized database from the MRI simulator for the specific MRI acquisition. Once the network was trained with volunteer-specific simulated MR data then it could be used (dashed lines) for generating T_1_ maps from MOLLI images acquired by the scanner

The overall processing with the personalized training neural network for T_1_ mapping with MOLLI is shown in Fig. 1. ECG triggers from the volunteer while acquiring the imaging data in the MRI scanner, along with pulse sequence parameters, were utilized by the Bloch simulator to generate a database of simulated MOLLI signals for an extended range of physiological native T_1_ and T_2_ values. For each MOLLI experiment on the scanner, a PTTN was trained with the acquisition-specific database. T_1_ maps were created on a pixel-by-pixel basis from the MOLLI images by applying the PTNN instead of the standard fitting methods.

The motivation behind using a neural network was to present an alternative to standard look-up methods, which had the potential to operate faster. The input to the neural network was signal intensities for a given pixel across images acquired with different TI values. The output of the neural network was the T_1_ of the given pixel. Ground truth values were the T_1_ values used for creating the simulated datasets by means of the Bloch equations and were used for training the neural network.

### CMR MOLLI protocol

MOLLI pulse sequences at a 1.5T Philips Achieva scanner (Philips Healthcare, Best, Netherlands) with two different acquisition schemes were used:

1. 5(3)3, the clinical MOLLI pulse sequence for native myocardial T_1_ mapping with an additional three heartbeat pause between inversions.
2. 5(0)3, a MOLLI pulse sequence without an additional pause between inversions, for mapping T_1_ faster.

All MOLLI pulse sequences follow the SQUAREMR protocol as previously described [19]. In more detail, a 4.74 ms hyperbolic secant adiabatic IR pulse was used with a bSSFP readout of 490 µs and a symmetric 3 lobe sinc shaped RF pulse (time-bandwidth product 4), 6 mm slice thickness, 35° excitation flip angle, rBW 1612.9 Hz/pixel, FOV 272 mm×272 mm, acquisition matrix 124 × 124, linear k-space trajectory and SENSE acceleration factor of 2. Prior to the bSSFP readout, 10 linear ramp up preparation pulses were used to reach steady state. All MOLLI pulse sequence intervals were defined in terms of heartbeats (i.e., not seconds).

### Signal simulation

Fast personalized network training was performed with volunteer-specific custom designed databases of MR signals derived from a Bloch simulator that not only took into account the pulse sequence and its parameters as executed by the scanner but also the actual ECG trigger points from the volunteer’s ECG. In other words, the specific RR intervals were used in the simulation database with the exact inversion times (TIs) of the single-shot image acquisitions at end-diastole. The TIs for each MOLLI experiment are not known a priori and depend on the actual RR intervals of the volunteer at the time the single-shot images are acquired. As a result, T_1_ estimation on a pixel-by-pixel basis for each experiment utilized a customized simulation database and a dedicated personalized network, which was trained with this database.

PTNN training was done with simulated MR signals under the premise that signals from simulated tissues (with specific T_1_ and T_2_ properties) would be similar to MRI scanner signals obtained from a phantom or volunteer (with the same T_1_ and T_2_ properties) if the same pulse sequence were applied [19]. Thus, a database of simulated MOLLI signal intensities was created for an extended range of physiological combinations of native T_1_ and T_2_ relaxation time constants. The MRI simulator processed signals as complex numbers [36]. In the end, magnitude values were extracted from the simulator for the neural network training because magnitude images were obtained from the MOLLI sequence in the scanner. MRI simulated MOLLI magnitude signal intensities in the database were normalized by dividing each one of them by the norm of all signal intensities.

The Bloch equation simulation platform CoreMRI [35–37, 40] was used. In brief, the platform simulates MR physics and exploits parallel computing with GPUs for high performance. For each MOLLI experiment, the MRI scanner pulse sequence was simulated taking into account the actual ECG triggers of the volunteer. The same methodology was used as previously described [18, 19, 41]. Simulations used T_1_ and T_2_ values as described earlier. Unrealistic combinations of T_1_ and T_2_ were excluded (e.g. T_1_ < T_2_, T_2_ < 20ms). The resulting simulated MOLLI signal intensities formed a customized database for the specific volunteer and the specific acquisition.

More specifically, the concept of the simulation platform was based on applying the Bloch equations on anatomical models of 21 spins along the slice direction so as to incorporate realistic slice profiles. The solutions of the Bloch equations resulted in the temporal evolution of each spin magnetization during the pulse sequence. A database size of 533,000 entries was used in four MOLLI 5(3)3 and four MOLLI 5(0)3 experiments. To further investigate PTNN performance with less training time, a smaller database of 21,560 entries was used in six MOLLI 5(3)3 experiments. Combinations of T_1_ and T_2_ values within the range of 600–2000 ms and 20–400 ms respectively, with a step of 1 ms or 5 ms were used to form a customized MOLLI signal database of either 21,560 (for simulations using a 5 ms step) or 533,400 (for simulations using a 1 ms) simulated intensities for each experiment to train the PTNN. Ten instances of Gaussian noise [42] were added to simulated data with signal-to-noise ratio SNR = 40 [6] for more realistic simulations. With noise augmentation, the final size of the training dataset was 215,600 and 5,334,000 respectively.

The effect of imperfect slice selection was simulated with the placement of 21 isochromats with identical magnetic properties along the slice selection direction. An odd number of isochromats was chosen to ensure that the central isochromat remained on-resonance with the RF pulse. The total of 21 isochromats was selected based on an optimized simulation parameter set from previous studies [41].

Since the Bloch simulator generated signals at each time step of the pulse sequence, relaxation effects during RF pulse application (both adiabatic inversion and excitation) were explicitly accounted for. In this study, the RF pulses were always simulated with a step of 5µs.

### PTNN architecture

A deep densely connected neural network for estimating T_1_ values from MOLLI images on a pixel-by-pixel basis was implemented from scratch. MR pixel intensities from eight MOLLI images [either 5(3)3 or 5(0)3] were input to the network (i.e., with an input layer of eight nodes). The architecture of the neural network consisted of three fully connected hidden layers of 40, 20, 8 nodes in each layer. A rectified linear unit (ReLU) activation function along with an adaptive moment estimation (Adam) stochastic gradient descent optimization algorithm were applied. The learning rate of the optimization algorithm was set to 0.001. The exponential decay rate for the first moment estimates was set to 0.6 and the exponential decay rate for the second moment estimates was set to 0.95. All hyper-parameter tuning was empirically optimized for a trade-off between estimations and overfitting evasion. Hyperparameters were tuned empirically once and applied for training the neural network for all subjects. No overfitting issues were identified. Also, the performance with testing data was measured for multiple sets of simulated data, where the ground truth values were known. This also suggested that there was no need to retune the hyperparameters individually for each subject. The PTNN was implemented in Python [43] using Keras [44] over Tensorflow [45].

The basic architecture of the PTNN was chosen considering that MOLLI T_1_ estimation is mainly a sequential arithmetic problem. Given 8 numbers (i.e. the samples from the inversion recovery curve), the neural network learns to predict one estimation, (i.e. the T_1_ value). Densely connected architecture is the simplest choice among other architectures that are more complex, time consuming with potential ambivalent performance.

### PTNN training

The database was randomly split into datasets of 70% training, 28% validation and 2% testing of the whole dataset for better optimization control. The mean-squared error loss function was used to minimize the error between the estimated and ground truth T_1_ values. A batch size of 20 inputs and 100 epochs were used to train the network.

Repeatable consistent determinism [46] in results obtained with neural networks is a desirable property in mission and safety critical systems, as CMR is used for diagnostic purposes. Thus, critical code aiding repeatable information assurance in the network learning sessions was incorporated [46]. This included setting real-time priority for uninterrupted task scheduling of sessions and seeded random number initializations in all neural network learning sessions.

### PTNN phantom validation

**Fig. 2:**
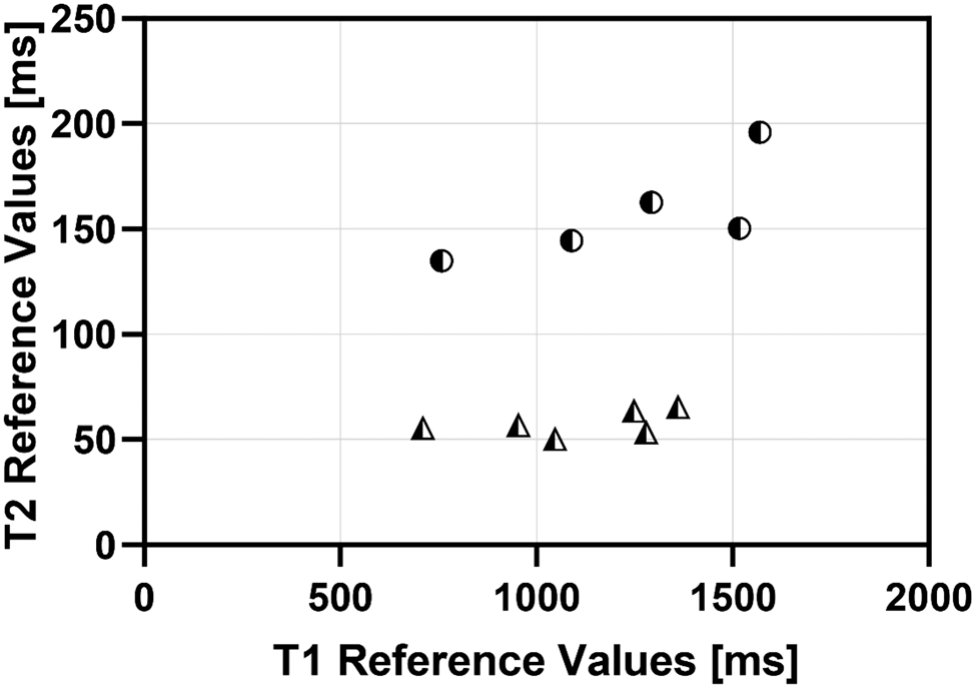
Reference T_1_ and T_2_ values of the 11 phantoms as measured by the reference methods. Phantoms simulating the blood with high T_2_ values are marked with semi-solid circles. Phantoms simulating the myocardium with low T_2_ values are marked with semi-solid triangles

The performance of the proposed PTNN method was evaluated with two sets of MOLLI experiments with eleven phantoms in total, as described in [19]. In brief, the phantoms were made of agar [47], CuSO4 and distilled water. The concentration of agar was titrated to change T2. The concentration of CuSO4 was titrated to change T1. The first set consisted of six phantoms with low T2 values (50–65 ms) and the second set consisted of five phantoms with high T2 values (135–200 ms), as shown in Fig. 2. For both sets T1 values covered the range from 700 to 1600 ms [19]. A simulated ECG of 60 bpm was used to trigger the MRI scanner MOLLI experiments and to train the PTNN with the Bloch simulator.

Phantom reference relaxation constants were measured with saturation-recovery for T_1_ values (Tsat = 0.01–15 s, TR = 25s) and with T_2_-prepared SSFP for T_2_ values since it has been already validated against slow spin-echo acquisitions [16].

### PTNN in vivo validation

MOLLI images from the MRI scanner were input to the PTNN to yield estimates of T_1_ on a pixel-by-pixel basis. For comparison, MOLLI T_1_ values with the conventional post processing method (“conventional fitting”) were calculated from the MOLLI magnitude images with a 3-parameter nonlinear least-squares curve fitting using the Levenberg-Marquardt algorithm [48–50]. Levenberg-Marquardt is considered a robust and standard algorithm for nonlinear least-squares routines [50] and was also applied in previous MOLLI T_1_ mapping studies [48]. Parameters were initialized by the standard MATLAB function for fitting with the Levenberg-Marquardt algorithm. Conventional fitting was implemented in MATLAB [51].

Normal volunteer experiments were conducted with written consent based on protocol approval by the local ethics committee. Performance of the proposed method was evaluated with MOLLI 5(3)3 in 10 healthy volunteers with no history of cardiac disease (10 men, age 34 ± 12 years). Moreover, MOLLI 5(0)3 was evaluated in four of the ten normal volunteers.

### Image analysis and statistics

T_1_ maps were generated with the PTNN on a pixel-by-pixel basis. T_1_ maps were also generated based on conventional T_1_ fitting. In phantom studies T_1_ values were measured with a rectangular ROI estimation in the center of each phantom T_1_ map. In vivo myocardial segmentations were performed manually. In healthy volunteer studies, left ventricular myocardium was segmented in the mid-ventricular short axis slice. Left ventricular blood T_1_ values were measured from a ROI in the left ventricular blood pool. Only one mid-ventricular slice per volunteer was used to ensure independent samples and minimize partial volume effects influencing the analysis.

Phantom T_1_ estimates from PTNN and conventional fitting methods were compared using Pearson’s correlation and modified Bland-Altman [52] plots to demonstrate the variance and agreement of the two methods with the reference standards. In normal volunteer studies, all estimated T_1_ values were reported as mean ± standard deviation.

For statistical analysis, student’s two tailed t-test for paired data was performed and a *p*-value less than 0.05 was considered statistically significant. Values are reported as mean ± SD.

## Results

### Phantom studies

The reference T_1_ and T_2_ values of the phantoms are shown in Fig. 2. T_1_ and T_2_ reference values ranged between 600 and 1600 ms and 50–200 ms respectively.

Measured T_1_ values obtained with the proposed PTNN method and the conventional fitting method, as well as the standard reference values are reported in Table 1. The proposed PTNN method showed lower T_1_ percent error across all phantoms of 2.34%±2.70% as compared to 5.55%±2.87% reported by the conventional measurements (*p* < 0.001, *N* = 11).

**Table 1:**
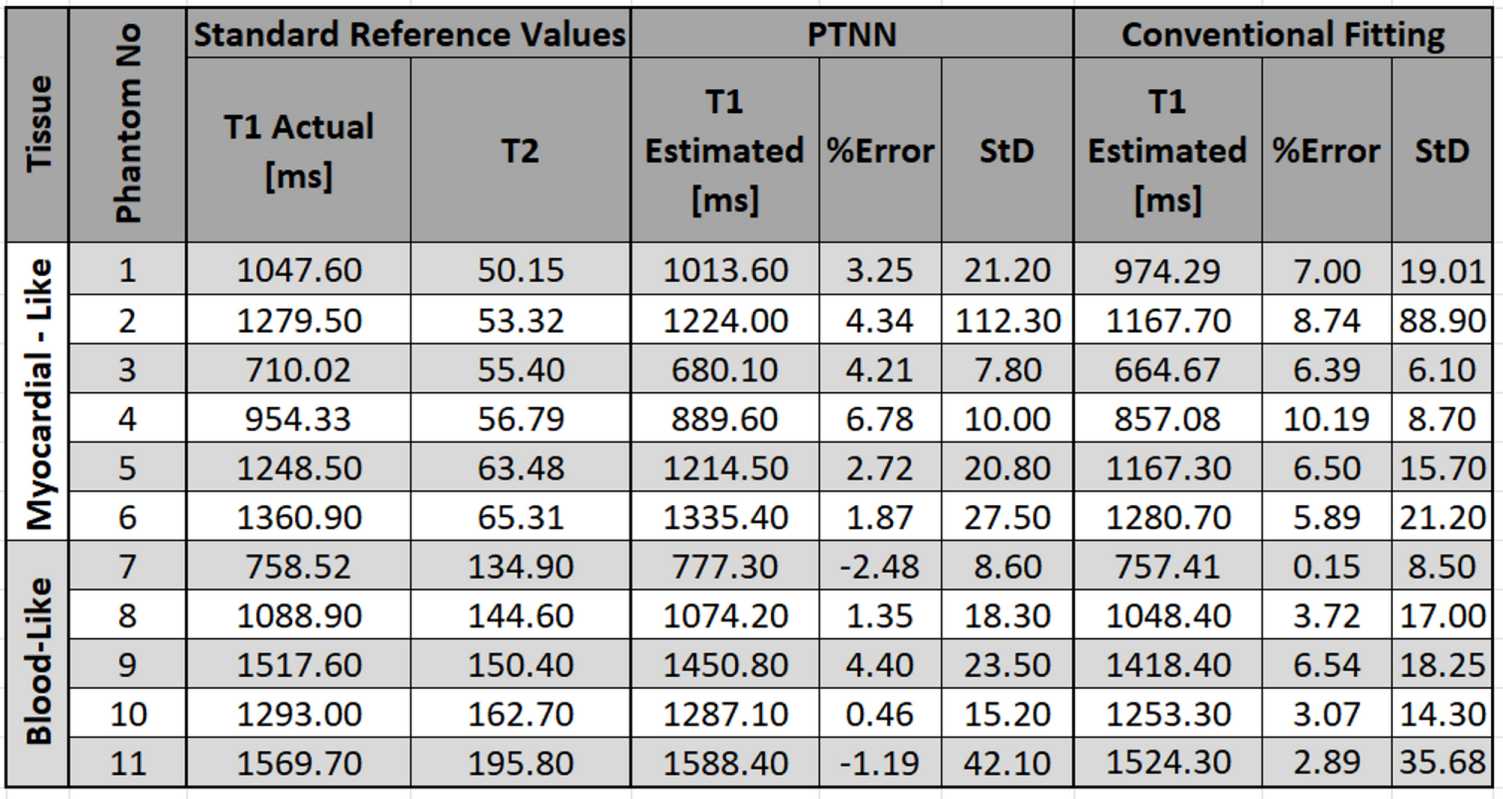
T_1_ measurements with PTNN and with the conventional fitting in phantoms and standard reference values. values.presented are mean, standard deviation across each phantom. %Error was calculated as (T_1_ actual – T_1_ Estimated)/T_1_ actual for each one of the two methods

**Fig. 3:**
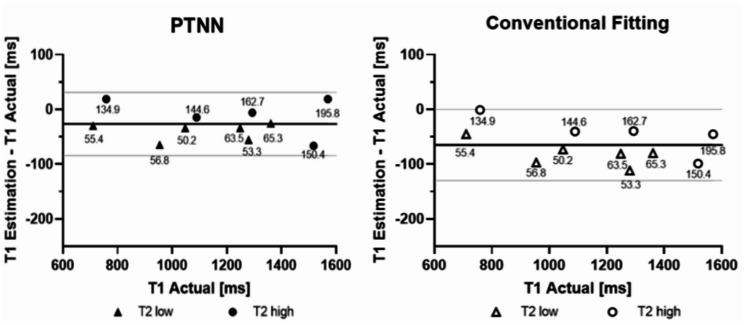
The PTNN approach yielded smaller bias compared to conventional fitting in 11 phantom experiments with MOLLI 5(3)3. Modified Bland Altman plots (thick horizontal lines represent the mean, grey lines represent the 95% confidence interval) for both methods are presented: PTNN top, solid signs and conventional fitting bottom, open signs. “Myocardium” phantoms with T_2_ < 100 ms are marked with triangles; “blood” phantoms with T_2_ > 100 ms are marked with circles. T_2_ values in ms are noted beside each symbol

Agreement between reference T_1_ values against either T_1_ estimates obtained with PTNN or T_1_ estimates obtained with conventional fitting are shown in Fig. 3. The agreement between T_1_ reference values and those obtained with the PTNN yielded a bias of -26.69 ms and 95% of differences lay between upper 31.15 ms and lower − 84.53 ms limits (-26.69 ± 29.51 ms) (*p* = 0.01, *N* = 11). Agreement between T_1_ reference values and conventional fitting values yielded a lower bias of -65.0 ms and 95% of differences lay between a wider interval of upper and lower limits 0.17 ms, -130.17ms respectively (-65.0 ± 33.25 ms), (*p* < 0.001, *N* = 11).

**Fig. 4:**
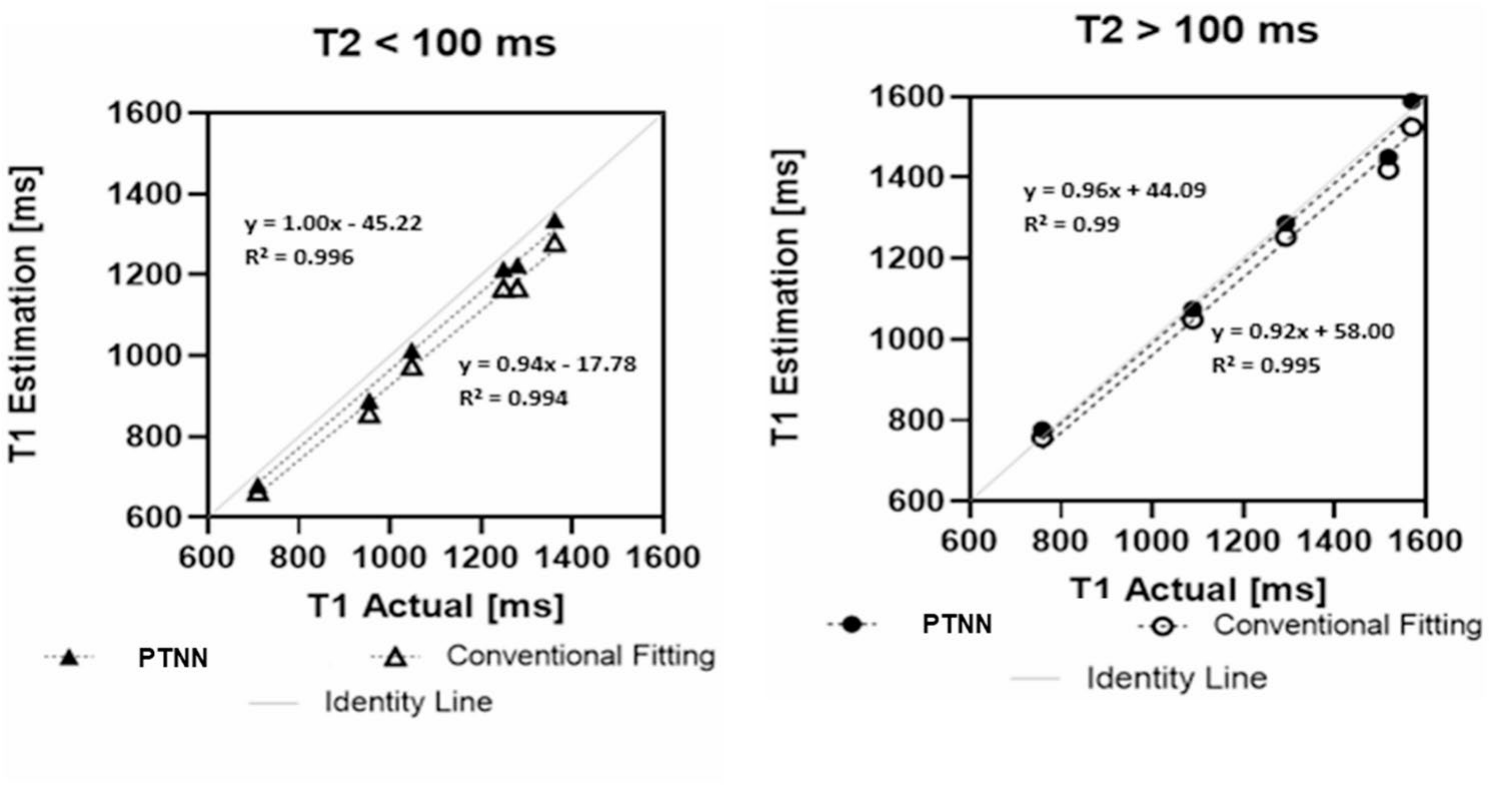
Correlation plots in phantoms between reference T_1_ values and those obtained with PTNN and conventional fitting for 11 phantom experiments with MOLLI 5(3)3. Less deflection from the line of identity is seen for the estimated PTNN T_1_ values (solid signs) compared to the estimated conventional fitting T_1_ values (open signs) for both “myocardium” phantoms with T_2_ < 100 ms (top) and “blood” phantoms with T_2_ > 100 ms (bottom)

Correlation plots (Fig. 4) show that in phantoms with low T_2_ values (top panel, e.g. myocardium, T_2_ < 100 ms) conventional fitting T_1_ estimates (open symbols) deviate from the actual T_1_ values (slope 0.94, intercept − 17.78, R^2^ = 0.99) whereas for phantoms with high T_2_ values (bottom panel, e.g. blood, T_2_ > 100ms) conventional fitting T_1_ estimates (open symbols) are closer to the actual T_1_ values (slope 0.92, intercept 58.00, R^2^ = 0.99). Similar results have previously been reported by Kellman [16]. The PTNN approach (solid symbols) yielded T_1_ estimates closer to the actual T_1_ values in both types of phantoms irrespective of the underlying T_2_ value (for T_2_ < 100 ms, top panel: slope 1.00, intercept − 45.22, R^2^ = 0.996 and for T_2_ > 100 ms, bottom panel: slope 0.96, intercept 44.09, R^2^ = 0.99).

### Human studies

**Fig. 5:**
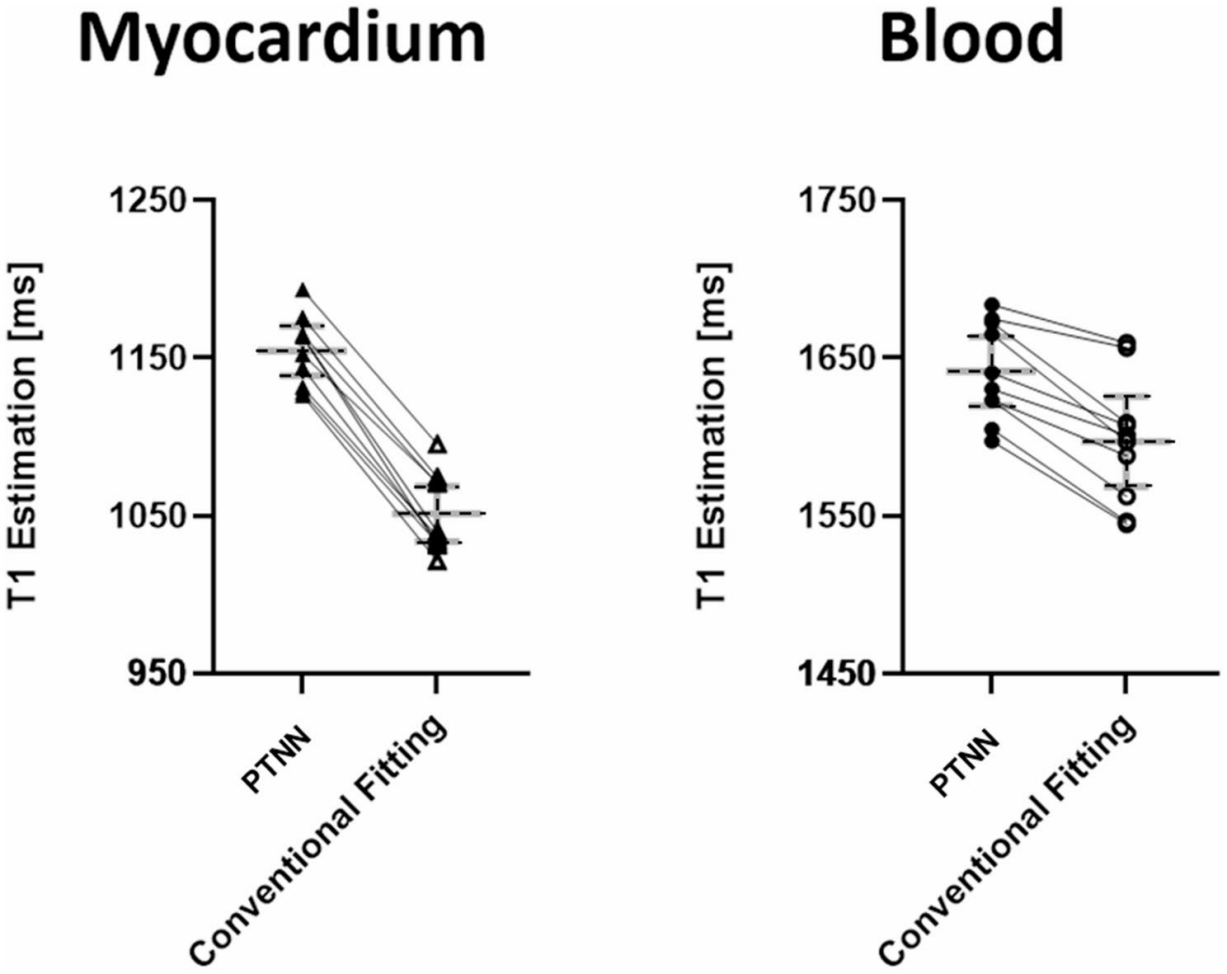
T_1_ estimates and error bars in 10 healthy volunteers for left ventricular myocardium (left) and left ventricular blood pool (right). A higher myocardial T_1_ mean for PTNN was observed compared to the conventional fitting. A higher blood T_1_ mean for PTNN was observed compared to the conventional fitting. T_1_ estimations as derived for each method are shown with black linking lines for each one of the healthy volunteers for both myocardium and blood

Inferior septal myocardial and left ventricular blood pool T_1_ estimates obtained by PTNN and conventional fitting for each of the ten healthy volunteers are presented in Fig. 5. The heart rate range in the volunteers was 53–73 bpm (60.3+/-5.2) which may not always allow for full magnetization recovery even with the longer R-R intervals. Conventional fitting T_1_ estimates were spread around a lower average T_1_ value (1050.8 ± 24.7 ms) compared to myocardial T_1_ estimates derived from PTNN, which were clustered close to a higher average T_1_ value (1152.4 ± 25.8 ms). Myocardial T_1_ estimates with PTNN were statistically significantly higher compared to those with conventional fitting (*p* < 0.05, *N* = 10). In the left ventricular blood pool, T_1_ estimates with PTNN yielded a slightly lower spread of values around a higher average T_1_ value (1640.7 ± 30.6 ms) compared to conventional fitting (1597.2 ± 39.9 ms). Blood pool T_1_ estimates with PTNN were statistically significantly higher compared to those with conventional fitting (*p* < 0.05, *N* = 10).

**Fig. 6:**
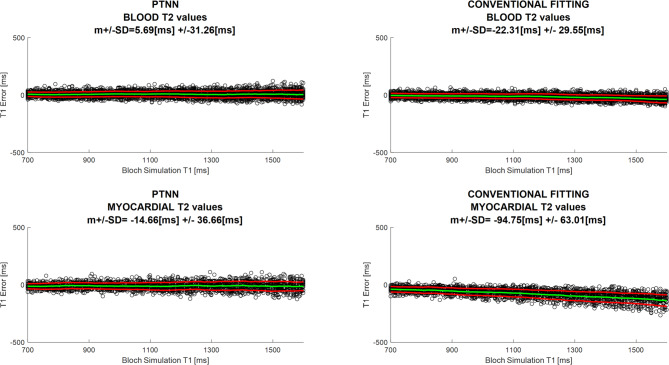
T_1_ estimated error vs. ground-truth Bloch Simulation values for SNR = 40. PTNN results are presented on the left compared to the conventional fitting method on the right for both blood (top) and myocardial (bottom) T_2_ values

One case where estimated T_1_ values are compared against the ground-truth i.e. T_1_ values used to create the data by means of the Bloch simulations is shown in Fig. 6. The T_1_ error is shown for both blood and myocardial T_2_ values. This is done for both PTNN and the conventional fitting method. PTNN showed lower mean T_1_ error and SD, as well as no underestimation for longer T_1_ values, compared to the conventional fitting method.

**Fig. 7:**
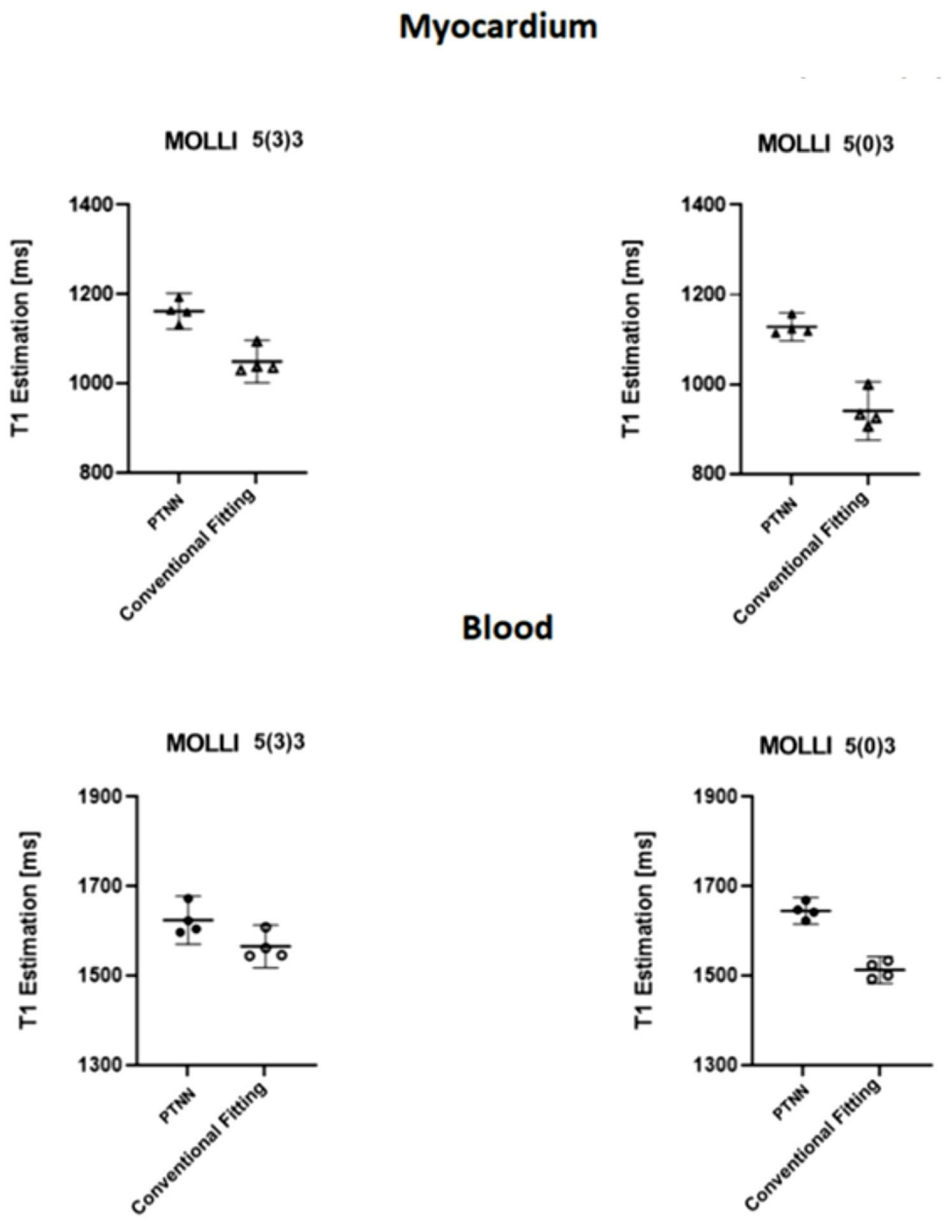
The effect of removing the recovery pause between MOLLI segments is shown in four normal volunteers. Overall higher T_1_ values were estimated with PTNN compared to the conventional fitting T_1_ for both myocardium (top) and blood (bottom), for both MOLLI schemes i.e. 5(3)3 (left column) and 5(0)3 (right column)

Fig. 7 shows the effect of removing the recovery pause between the effect of removing the recovery pause between MOLLI segments for PTNN and conventional fitting in both myocardium and blood in four healthy volunteers. Myocardial T_1_ PTNN estimates were clustered to a higher T_1_ value compared to T_1_ conventional fitting for both the 5(3)3 scheme (1162.2 ± 19.7 ms with PTNN vs. 1050.9 ± 29.8 ms with conventional fitting, *p* < 0.05) and the 5(0)3 scheme (1127.1 ± 19.7 ms with PTNN vs. 940.6 ± 41.03 ms with conventional fitting, *p* < 0.05). In blood, T_1_ PTNN estimates were clustered higher compared to T_1_ conventional fitting for both the 5(3)3 scheme (1624.7 ± 33.9 ms with PTNN vs. 1565.6 ± 29.9 ms with conventional fitting, *p* < 0.05) and the 5(0)3 scheme (1645.4 ± 18.7 ms with PTNN vs. 1513.0 ± 19.0 ms with conventional fitting, *p* < 0.05).

**Fig. 8:**
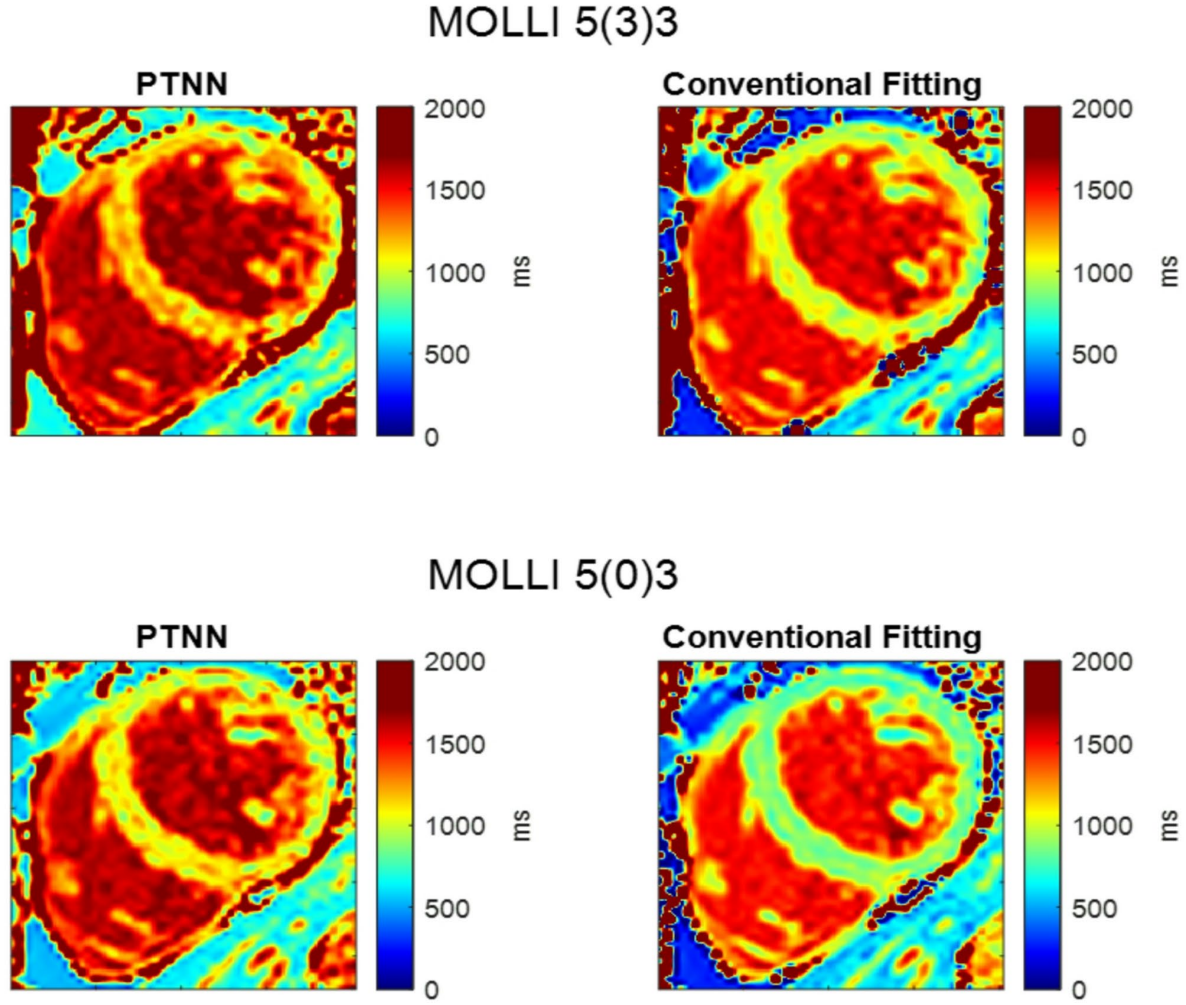
T_1_ maps of a healthy volunteer experiment with MOLLI 5(3)3 (top) and 5(0)3 (bottom) acquisition schemes. T_1_ maps derived from PTNN (left) showed visually higher T_1_ values both in the myocardium and in the blood compared to the conventional fitting T_1_ map (right). The color scale is based on the quantitative MRI study group [53]

Fig. 8 shows T_1_ maps derived from PTNN and the conventional fitting method for both 5(3)3 and 5(0)3 acquisition schemes in a healthy volunteer.

The overall PTNN training time for each human experiment was 128s when using 215,600 entries in the simulation database and 1100s when using 5,334,000 entries in the simulation database. This was accomplished on a laptop CPU. Using dedicated hardware with multiple GPU’s should enable clinically useful computation times.

**Fig. 9:**
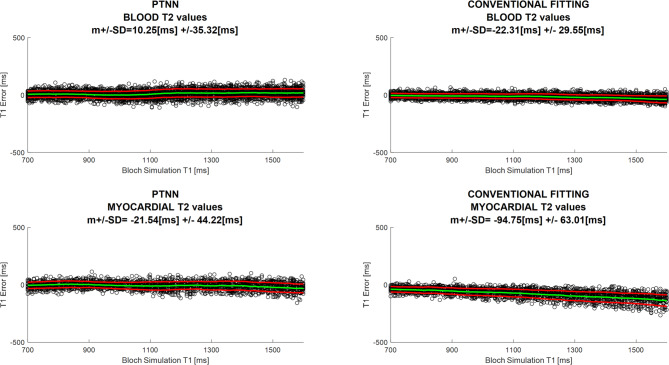
T1 estimated error vs. ground-truth values (SNR = 40) for a smaller simulation database (_1_1,078 entries). PTNN results are presented on the left compared to conventional fitting method on the right for both blood (top) and myocardial (bottom) T_1_ values. (The testing dataset used for Fig. 6 was similar in size to that used for Fig. 9 so that it is easier to compare the two figures)

**Fig. 10:**
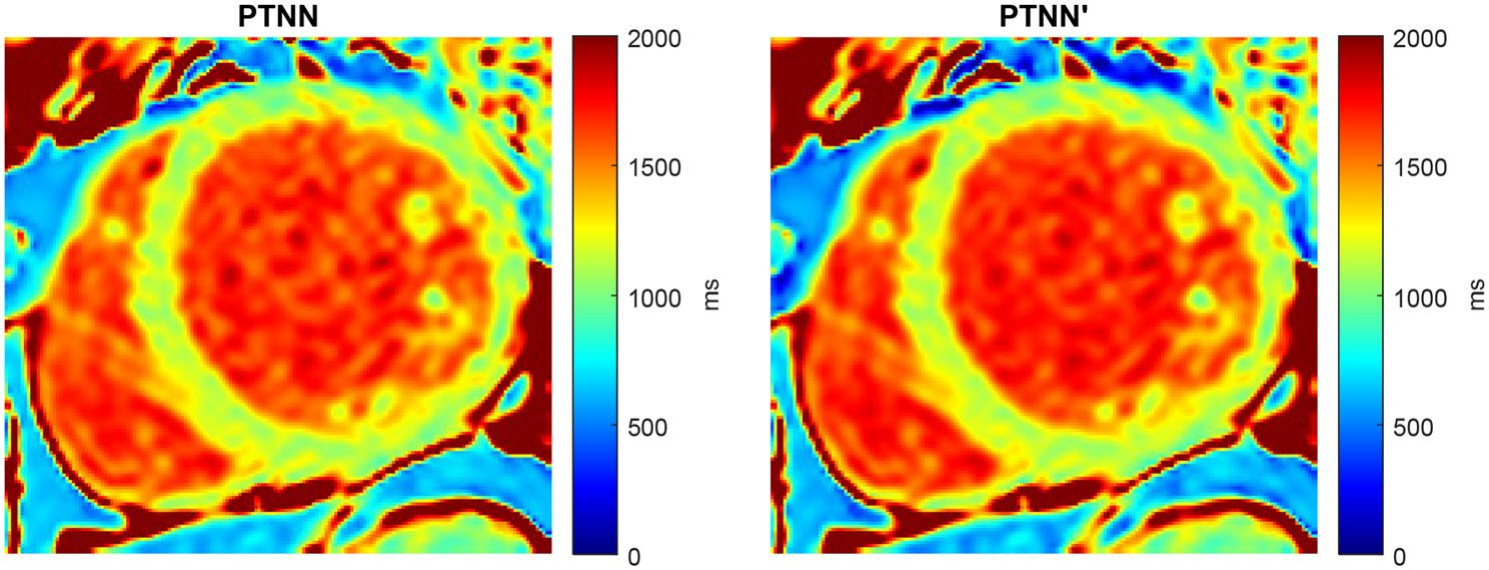
T1 maps estimated by the PTNN. The left T_1_ map used a large simulation database (21,560 entries) whereas the right one used a smaller simulation database (1,078 entries). No visible differences can be observed

Figure 9 shows one case where estimated T_1_ values were compared against the ground-truth for simulation data generated with a larger step, of 100ms. This means less time needed for the simulator to create a small database of only 1,078 entries instead of 21,560. In this case, 35s was the time needed to train the network instead of 128s for the large database (in both cases datasets were augmented 10 times by injecting different noise levels). In the dataset of 320 × 320 pixels, the proposed PTNN method took approximately 47s (including simulation database generation, NN training, and NN execution), while SQUAREMR reported 2 m:12s (including simulation database generation and lookup-table execution). Similar results were observed with the small database (Fig. 9) as with the larger one (Fig. 6). Similar results were also observed for the resulting MOLLI 5(3)3 T_1_ maps from the small simulation dataset (Fig. 10) and the larger dataset (Fig. 8). The faster speed of the PTNN was made possible due to the smaller simulation database that could be used with PTNN because of its ability to interpolate between lookup-table entries.

## Discussion

A personalized approach to training neural networks with simulated data was presented for estimating T_1_ maps from a clinical MOLLI pulse sequence. The use of a deep learning neural network that was trained specifically for each volunteer with simulated signal intensities, taking into account the pulse sequence with its parameters as well as the volunteer’s recorded ECG triggers, yielded overall higher T_1_ estimates than conventional fitting both with the clinical MOLLI 5(3)3 as well as with the proposed faster MOLLI implementation 5(0)3, which reduced acquisition to eight heartbeats, by mitigating the dependence on the patient’s heart rate variations.

Several studies have already shown that MOLLI with conventional fitting underestimates T_1_ [16, 54]. Moreover, T_1_ measurement error is influenced by several acquisition parameters [16]. The proposed PTNN is based on simulations of the actual pulse sequence that runs on the MRI scanner. The concept is that signals from simulated tissues with specific T_1_ and T_2_ properties would be similar to the signals obtained from the MRI scanner from real tissues with the same relaxation properties, assuming that the same pulse sequence was applied. The proposed PTNN was trained with simulated signals and yielded higher T_1_ estimates against the conventional MOLLI approach for a range of physiological T_2_ values (in phantoms) and heart rates (in volunteers).

Recent cMRF [34] studies have shown higher mean T_1_ myocardial values (for 15 heartbeats: 1043 ms direct matching MRF and 1044 ms DIP MRF; for 5 heartbeats: 1065 ms direct matching MRF and 1035 ms DIP MRF) compared to MOLLI conventional fitting (1006 ms). To reiterate, the PTNN method yielded a mean T1 value of 1150 ms with 5(3)3 and 1127 ms with 5(0)3 compared to MOLLI conventional fitting (1050 ms).

There was a consistent trend in both phantom and volunteer results i.e. higher values obtained by PTNN. As was already shown in Table 1, for the phantom experiments, higher values were obtained with PTNN compared to conventional fitting. For healthy volunteer experiments, higher values were also obtained with PTNN compared to conventional fitting (Figs. 5 and 7). Comparing values obtained with MOLLI against standard reference values is a different issue. This could only be done in phantoms where such standard reference values existed. In these phantom experiments, underestimation was seen with MOLLI irrespective whether PTNN or conventional fitting was used, albeit less of underestimation was seen when PTNN was used.

Results of phantom experiments demonstrated smaller bias in Modified Bland Altman plots (Fig. 3), compared to the conventional post-processing MOLLI curve fitting. As PTNN phantom T_1_ estimates were of higher value compared to conventional fitting estimates and comparable to reference standard values. PTNN in phantom experiments was trained with a simulated ECG trigger of 60 bpm. Same in healthy volunteers, PTNN was trained with volunteer specific heart rates and higher T_1_ estimates with similar precision was presented in comparison to conventional fitting methods. Experiments on healthy volunteers showed higher T_1_ values and similar precision not only with the MOLLI 5(3)3 but also with MOLLI 5(0)3, which is a faster MOLLI protocol without a pause between modified–Look-Locker inversions, allowing for native myocardial T_1_ characterization in just eight heart beats. This could be beneficial while scanning patients with limited breath-holding ability.

T_1_ estimates with PTNN yielded higher values for native myocardium (1152.4 ± 25.8 ms) when compared to conventional MOLLI post-processing (1050.8 ± 24.7 ms), as shown in Fig. 5. T_1_ myocardial estimates with PTNN were found closer to standard reference SASHA T_1_ values 1170 ± 27.0 ms [16]. Similarly, higher T_1_ values were found with PTNN in blood (1640.7 ± 30.6 ms) compared to conventional fitting post processing (1597.2 ± 39.9 ms). T_1_ blood estimations with PTNN were found closer to standard reference SASHA T_1_ values 1639 ± 97 ms [16]. Conventional MOLLI T_1_ values estimated in this work were similar to MOLLI T_1_ values reported in the literature: 1052 ± 41 ms [16] for native myocardium T_1_ and 1534 ms [55] for blood T_1_. SASHA is considered more accurate than MOLLI, despite having a reduced dynamic range, because each measurement is independent of the others as saturation preparation erases prior history [16].

With the faster MOLLI 5(0)3 scheme (Fig. 7) PTNN T_1_ estimation outperformed conventional post-processing for both myocardium (1127.1 ± 19.7 ms vs. 940.6 ± 41.03 ms) and blood (1645.4 ± 18.7 ms vs. 1513.0 ± 19.0 ms) demonstrating T_1_ values for both myocardium and blood similar to PTNN method values for standard MOLLI 5(3)3 scheme (i.e. 1162.2 ± 19.7 ms for myocardium and 1624.7 ± 33.9 ms for blood) and comparable to standard reference SASHA T_1_ values (i.e. 1170 ± 27.0 ms for myocardium and 1639 ± 97 ms for blood) found in literature [55].

The PTNN is trained every time on new data since the actual ECG is different for every experiment. Transfer learning, a method that reuses a model already trained on generic data, as well as partial pretraining, an architecture design where weights are partially initialized from a pretrained model, can be applied to improve performance or reduce training time on similar data from a related new task. Transfer learning or partial pretraining are widely applicable in the absence of an abundance of training data for a specific task. However, bias or overfitting may be introduced by dominating pretrained modules. If bias and overfitting can be avoided then PTNN could potentially utilize transfer learning options to further reduce training time.

T_1_ estimation with PTNN was not affected much by heart rate variations. For each human study, the actual RR intervals, which may vary with time, were provided to the neural network as input. As a result, native myocardial and blood T_1_ were estimated taking into account heart rate variations during the MOLLI acquisition. In other studies [28–30], simulated heart rate variations have been used for training the network instead of training with the specific pattern of variation obtained from each volunteer. With PTNN, fewer training datasets were required. Even in the case of shortest MOLLI experiments, higher PTNN T_1_ values with comparable precision were obtained in both myocardium and blood compared to conventional T_1_ estimates.

As expected [16], for tissues with low T_2_ values (e.g. myocardium) conventional fitting T_1_ estimates deviated from the actual T_1_ values whereas for tissues with high T_2_ values (e.g. blood), conventional fitting T_1_ estimates were closer to the actual T_1_ values. Further, MOLLI T_1_-estimates in the blood pool were not affected as much by the multiple SSFP readouts after each inversion due to blood wash-out between heart beats [16].

The PTNN was trained on MOLLI-based tissue signal intensities with an MR protocol that was designed to obtain T_1_ estimates that are not affected by the tissue’s T_2_ as much as possible. Therefore, PTNN T_1_ estimates from both the conventional fitting and PTNN were similarly affected by this confounder. This may explain why the error in T_1_ estimates for phantoms with long and short T_2_ times appeared to be similar between two methods (Fig. 3). Considering that T_2_ modulates the acquired signal, a modified MOLLI [18] with longer duration RF pulses and higher excitation flip angles could potentially be used to generate signal intensities with increased T2 values sensitivity for network training so that the PTNN learns how to compensate for different T2 values.

The use of a neural network trained with simulations from actual patient physiological data to improve MOLLI T_1_ estimates is a new concept. In the past T_1_ MOLLI estimates obtained with neural networks were reported to be similar to those obtained with conventional fitting MOLLI. This could potentially be attributed to training the network with patient data obtained with conventional fitting MOLLI maps, which are known to underestimate T_1_ [27, 28]. Prior published work that uses simulated data for training has also yielded T_1_ estimates comparable to those obtained with conventional fitting MOLLI. This may be explained because this prior work did not take into account the patient’s actual physiological data during training but was rather trained with a range of potential heart rates [28, 29]. More specifically, in MyomapNet, a fixed heart rate of 60 bpm was used in numerical simulation. In DeepBless and improved MyomapNet there is an attempt to introduce various heart rates with beat-to-beat variations in the numerical simulations. But even this attempt was within a limited scope, as all possible heart rate and beat-to-beat variations may not be included. With the proposed PTNN, which uses only the actual patient’s physiological data for training, higher T_1_ estimates were obtained compared to conventional fitting MOLLI methods for the first time without sacrificing precision. This was also seen with the faster MOLLI scheme (eight heart beats acquisition).

Our proposed scheme can be seen as an implicit neural network as it learns an implicit mapping from signal intensity measurements to T_1_ and T_2_ values. In our case we cover the entire range of expected T_1_ and T_2_ values as opposed to zero-shot or few-shot learning strategies which have the advantage that require much less training data but where the result may be harder to predict accurately.

As far as execution time is concerned, as stated above, PTNN time performance (i.e., 128s with 21.560 entries) is clinically applicable, taking also into account the greater computation power of MRI servers in practice. PTNN requires that the database of simulated signals be formed after the pulse sequence has run and the volunteer-specific ECG triggers have been recorded. Therefore, it is essential that the database is created within reasonable times so that the neural network can be trained and used to produce a T_1_ map for clinical use. As the use of online cloud-based MRI simulators is becoming more widespread, higher simulation speeds should be expected [41]. In the future, along with GPU-based acceleration, the generation of the database may only take a couple of seconds and could potentially be integrated in the clinical workflow as a routine application implemented within the Gadgetron reconstruction framework [56].

The simulation execution duration was 22s for the 21,560 database entries (5ms step) and 17m:47s for the 533,400 database entries (1ms step), on a server with Tesla C2075 GPU cards [19]. Also, the time needed to apply the PTNN was 1.9s for 320 × 320 pixels and 0.5s for 128 × 128 pixels, on a laptop CPU. Simulation data with a much larger step of 100ms can be used to create an even smaller database. In this case of 1,078 entries, the time needed to train the PTNN was only 35s instead of 128s that was required with the larger database of 21,560 entries. Therefore, considering that Figs. 9 and 10 appear similar, with PTNN there is no need for a large number of simulations since the neural network shows an ability to interpolate.

Compared to cMRF studies, PTNN also integrates the ECG timing in the simulated dataset and generates the corresponding simulated database for each scan. Both the recent framework of DIP-MRF and PTNN take into account ECG timings, slice profile and relaxation during the adiabatic excitation; however, PTNN is less computationally complex and faster based on published execution times [34]. On the other hand, the current cMRF framework produces both T_1_ and T_2_ maps, suppresses noise and aliasing artifacts. Interestingly, both DIP-MRF and PTNN result in higher T_1_ values compared to conventional fitting methods. Whereas PTNN is a postprocessing MOLLI method based on a clinically applicable sequence, cMRF is currently applied with custom research sequences not available in the clinics but remains a promising workflow for rapid multi-parametric tissue property mapping with the potential to improve MRI exam efficiency. However, cMRF still needs validation and standardization to be clinically adopted.

In this study some limitations apply. No variations of flip angle were taken into consideration. Possible variations of B_1_ should be considered including various flip angle values for MOLLI sequence in simulation datasets. However, we did not consider such variations because, as shown by Kellman et al. [16], the influence of the flip angle error due to B_1_ variations in the SSFP readout is about 2% in myocardial T_1_ estimation. This is also in line with this study’s phantom results that show no bias, especially at higher T_1_ values were errors would be more apparent. There was no experimentation on higher heart rates and on patient data.

It is expected that even in cases of extreme arrhythmias, where the depolarization of the LV has its origin at the SA node, PTNN would yield useful results. This is because the MOLLI acquisition is triggered by the R-wave and these timings are recorded and used by PTNN. However, in cases with highly irregular cardiac rhythms (e.g. LV premature beats, atrial fibrillation) it is likely that PTNN will not perform well. In such cases one would also expect compromised image quality since the MOLLI acquisition may not always coincide with diastole.

Noise was added to the simulated dataset for more realistic simulations with SNR = 40 according to the results shown in a work by P. Kellman [6] where experiments measured SNR close to 40 in ROIs of the septum and LV blood pool. Same ROIs are studied in present work. Nevertheless, different SNRs may apply in different ROIs.

Furthermore, a limited range of T_1_ values (600–2000 ms) was selected while generating data in simulator because that was the range of T_1_ values, T_2_ values of interest in the myocardium and blood pool in MOLLI experiments. Wider ranges of T_1_ values could be selected for better visual display of other tissues than myocardium, such as fat.

Phase Sensitive Inversion Recovery (PSIR), which was first used in conjunction with late gadolinium enhancement (LGE) [57] and was later applied to MOLLI T_1_ mapping [58], is based on phase sensitive image reconstruction and the preservation of polarity of the MR signal after inversion. This improves the quality of T_1_ maps due to the restored dynamic range and the normally distributed noise around zero. In principle, the PTNN approach is compatible with PSIR reconstruction and should be investigated in the future.

A potential limitation of this study was that no direct validation in phantoms was performed for PTNN with MOLLI 5(0)3 PTNN. Instead, MOLLI 5(0)3 was only compared to MOLLI 5(3)3 in normal volunteers.

## Conclusions

In conclusion, PTNN is a new post-processing method for MOLLI data. It utilizes the actual ECG trigger data from the MR examination, for generating simulated signals for training a neural network for each individual volunteer for yielding higher values in native T_1_ myocardial tissue and blood and achieving better accuracy in phantoms for a physiological range of T_1_ and T_2_ values. This allowed a faster MOLLI scheme without a pause to be applied in a series of healthy volunteer experiments with existing clinical pulse sequences without compromising accuracy.

## Data Availability

The datasets used and/or analyzed during the current study are available from the corresponding author on reasonable request.

## Abbreviations

bSSFP: Balanced Steady State Free Precession
FOV: Field of View
IR: Inversion Recovery
LV: Left Ventricle
MOLLI: Modified Look-Locker Inversion
ms: milliseconds
PTNN: Personalized Training Neural Network
rBW: Receiver Bandwidth
RF: Radiofrequency
ROI: Region Of Interest
SASHA: Saturation recovery Single-SHot Acquisition
SENSE: SENSitivity Encoding
SNR: Signal to Noise Ratio
SD: Standard Deviation
SQUAREMR: Simulations for QUAntifying RElaxation Magnetic Resonance
µs: Microseconds
